# Functional outcomes are preserved in adult acetabular dysplasia with radiographic evidence of lumbosacral spine anomalies: an investigation in hip-spine syndrome

**DOI:** 10.1186/s12891-022-05334-5

**Published:** 2022-04-25

**Authors:** Aaron Shi, Joshua Sun, Avneesh Chhabra, Uma Thakur, Yin Xi, Ajay Kohli, Joel Wells

**Affiliations:** 1 Department of Orthopaedic Surgery, University of Texas Southwestern Medical Center, 5323 Harry Hines Blvd, Dallas, TX 75390-8883 USA; 2 Department of Radiology, University of Texas Southwestern Medical Center, 5323 Harry Hines Blvd, Dallas, TX 75390-8883 USA

## Abstract

**Purpose:**

Acetabular dysplasia (AD) is a debilitating condition which results in impaired hip function, leading to hip-spine syndrome with anomalies identifiable on plain radiographs. However, no study to date has investigated the association between radiographic spine anomalies and functional outcomes in AD. We hypothesize that AD patients with radiographic evidence of lumbar spine anomalies are associated with decreased function in comparison to those without such radiographic findings.

**Patients and methods:**

One hundred thirty-five hips underwent a full four-view hip radiograph series, and two observers analyzed hip and spine variables using standard radiographs and obtained Castellvi grade, assessment of spondylolisthesis, and L4-S1 interpedicular distance. A comprehensive hip questionnaire was administered which included Harris Hip Score (HHS) and Hip Disability and Osteoarthritis Outcome Score (HOOS) to assess patient function. Correlations between HHS and HOOS and radiographic spinal measurements were calculated, and *p*-values were corrected for multiple comparison using the Holm’s method.

**Results:**

Out of 135 patients, 119 were female (88.1%) and 16 were male (11.9%). Average age of presentation was 34.2 years, and average BMI was 26. There was no statistically significant correlation between Castellvi grade, presence of spondylolisthesis, or L4-S1 interpedicular distance and the patient-reported outcome measures HHS or HOOS. Conversely, a significant correlation was observed between Femoro-Epiphyseal Acetabular Roof (FEAR) index and HOOS of the contralateral hip (correlation coefficient = 0.38, adjusted *p* = 0.03) and Tönnis angle of AD severity and HHS of the contralateral hip (correlation coefficient = − 0.33, adjusted *p* = 0.04).

**Conclusion:**

Severity of spinal anomalies measured by Castellvi grade and spondylolisthesis in patients with AD was not associated with decreased patient function in the ipsilateral diseased hip. To our knowledge, this is the first study to date to report the relationship between radiographically identifiable lumbosacral abnormalities and hip function in AD.

## Introduction

Acetabular dysplasia (AD), an unstable ball-in-socket hip joint with insufficient coverage of the femoral head by the acetabulum, is a debilitating condition with a prevalence between 5 and 13% in the general population [1, 2]. Assessment of hip radiographs is critical in diagnosis of AD and allow for targeted surgical treatment [3]. The current primary surgical management of AD involves hip preservation surgery or total hip arthroplasty (THA). Currently PAO serves as a strong alternative to total hip arthroplasty with 15-year postoperative hip survivorship of 92% and is indicated in younger patients (less than 40 years of age) with concentrically reduced hips and congruous joint space without end stage osteoarthritis [4, 5].

AD has been understood as a risk factor for pain and degenerative changes in the hip joint, leading to an improper hip-spine alignment or hip-spine syndrome, characterized by flexion deformity of the hip that rotates the pelvis forward and results in spine symptoms in many patients [6, 7]. This coexistence of hip and spine pathology has been previously described in patients with hip osteoarthritis, showing evidence of lumbar spinal stenosis, and in patients with femoroacetabular impingement (FAI), showing increased anterior pelvic tilt, decreased sagittal mobility, lower pelvic incidence, and increased lumbar lordosis with increased static kyphosis compared to healthy controls [8–11]. This relationship has been investigated in patients with AD as well. Previous studies in AD have noted lumbar hyperlordosis and increased pelvic incidence, hip extension, and internal rotation as associated factors to pathology, as well as smaller gluteus medius circumferences and changes in femoral morphology [12–15].

In a previous study, we have established the association between widened L4-S1 interpedicular distance and increased frequency and severity of Castellvi grade in up to 40% of patients with AD [16]. However, no study to date has examined the association between these radiographically-identifiable lumbosacral anomalies and patient function in AD. The purpose of this study is to investigate the relationship between hip function and radiographic evidence of spondylolisthesis or lumbosacral transitional vertebrae in patients with AD. Due to our understanding from our previous studies as well as our clinical experience with AD patients frequently presenting with complaints of both the hip and spine, we hypothesize that AD patients with radiographic findings of spine deformity will exhibit decreased functional outcomes compared to AD patients without such radiographic findings.

## Materials and methods

This was a cross-sectional study of a prospectively collected data registry which followed all Health Insurance Portability and Accountability Act regulations and obtained approval from local Institutional Review Board. All data collected in the registry is from one academic center. Informed consent was waived for retrospective evaluation.

### Patient selection

**Fig. 1:**
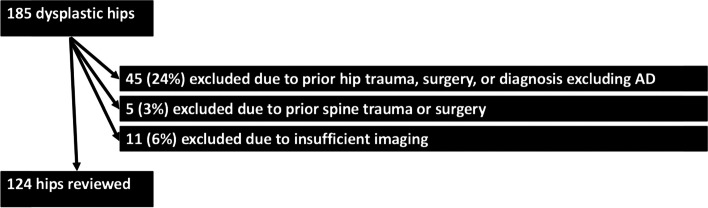
Inclusion and exclusion criteria for dysplastic hips

Analysis of our orthopedic hip preservation registry from the years 2016–2021 identified 185 hips (153 females and 32 males) who presented to our tertiary university orthopedic department with a chief complaint of hip pain and received a diagnosis of AD. Exclusion criteria involved patients with previous surgery or trauma to their hip, previous surgery or trauma to their spine, pre-existing medical condition affecting hip or spine mobility (e.g. Ehler’s Danlos, cerebral palsy), and insufficiency radiographic imaging (lack of full radiograph series of anterior-posterior and false-profile views, poor visibility of radiographs to accurately perform measurements, or skeletal immaturity assessed through the Risser stage with 4 or less considered immature [17]). This study includes the same patient cohort of our previous study [16]. This inclusion and exclusion criteria can be observed in Fig. 1.

### Radiograph analysis

All patients underwent standardized full four view radiograph series of the symptomatic hip. All measurements were performed on iSite (Philips, Best, Netherlands) software. Anterior-posterior pelvis and false-profile views of the bilateral hips as well as anterior-posterior and lateral views of the lumbar spine were assessed by an experienced hip preservation surgeon JW, and qualitative evaluation of spine anomalies was performed by two experienced musculoskeletal radiologists AC and UT according to standardized procedures for assessing pelvic and lumbar spine structures [18]. The false profile view was obtained with the patient’s pelvis rotated 65° and with the foot on the affected side parallel to the radiographic cassette. Additionally, the data collector AS was trained by the same hip surgeon and musculoskeletal radiologists to complete hip and spine quantitative variables, and the senior readers frequently verified correct measurement methods were used with random checks.

**Fig. 2:**
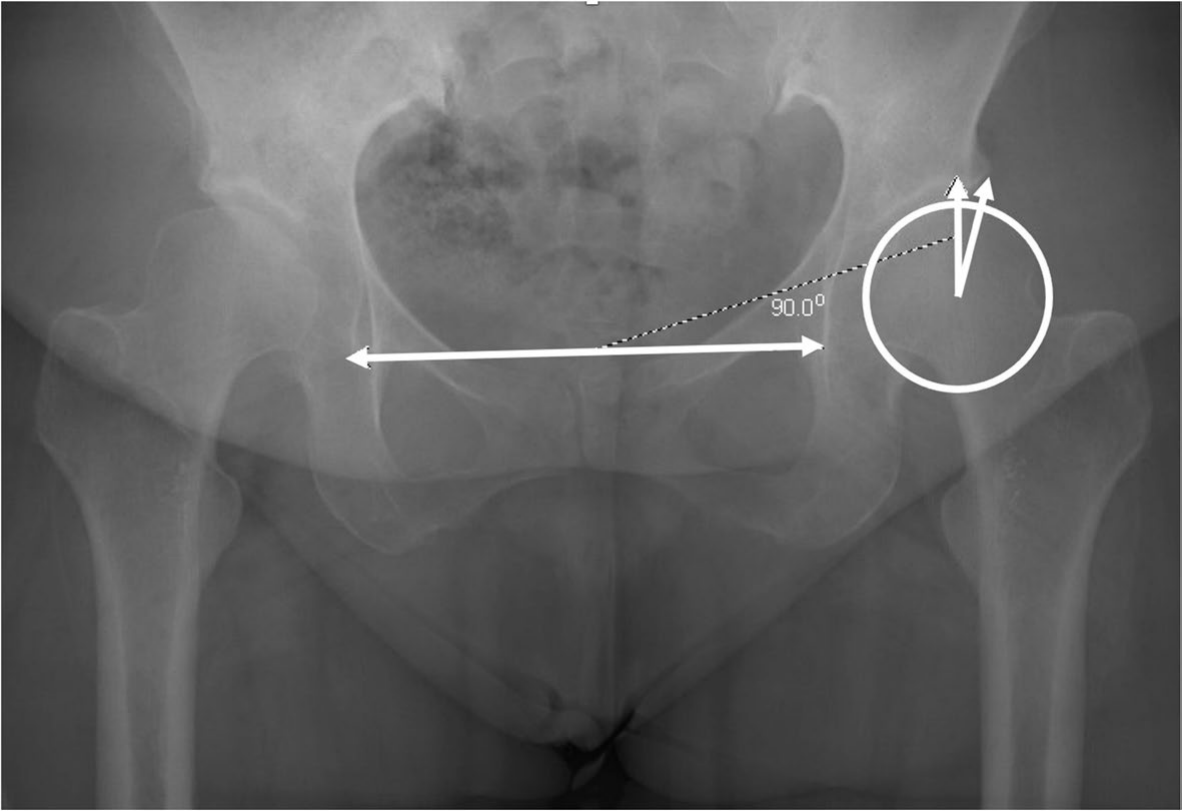
LCEA of 15.4° suggestive of AD. Angle is drawn perpendicular to transverse pelvic axis and centered on femoral head

**Fig. 3:**
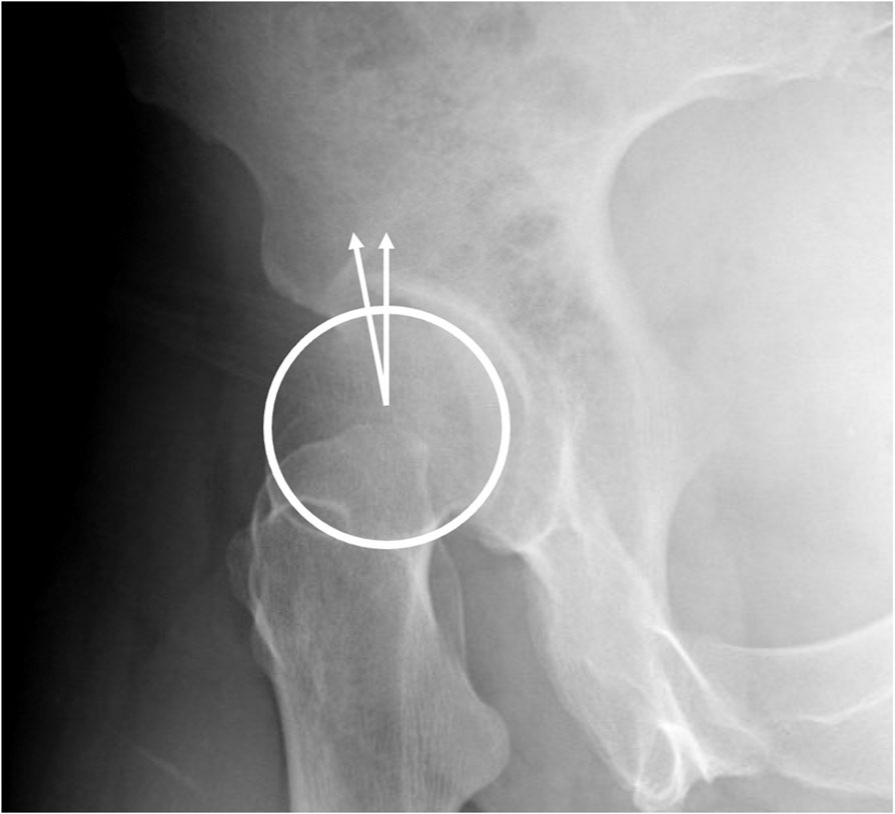
ACEA of 10.2° suggestive of dysplasia. Angle centered at femoral head with one vertical arm and another arm at most anterior portion of acetabular sourcil

**Fig. 4:**
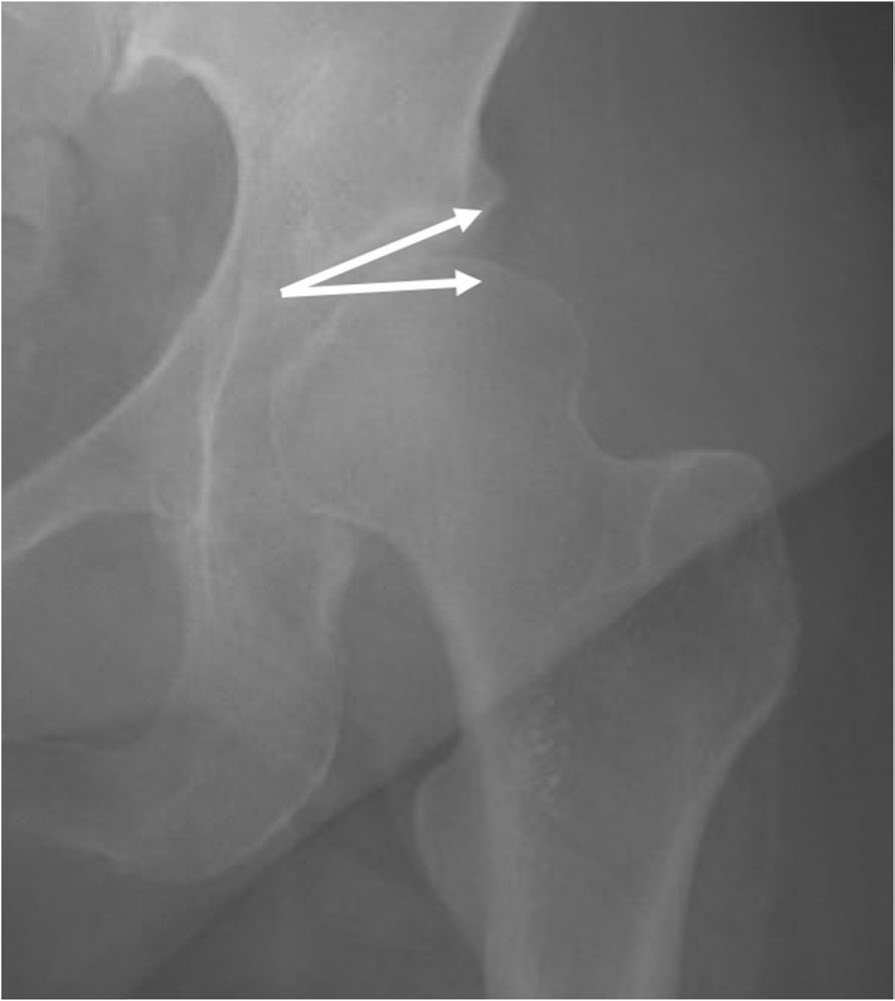
Tönnis angle of 20.8° suggestive of AD. An angle whose base is parallel to transverse pelvic axis and connects the most inferior and superior portions of the sourcil

**Fig. 5:**
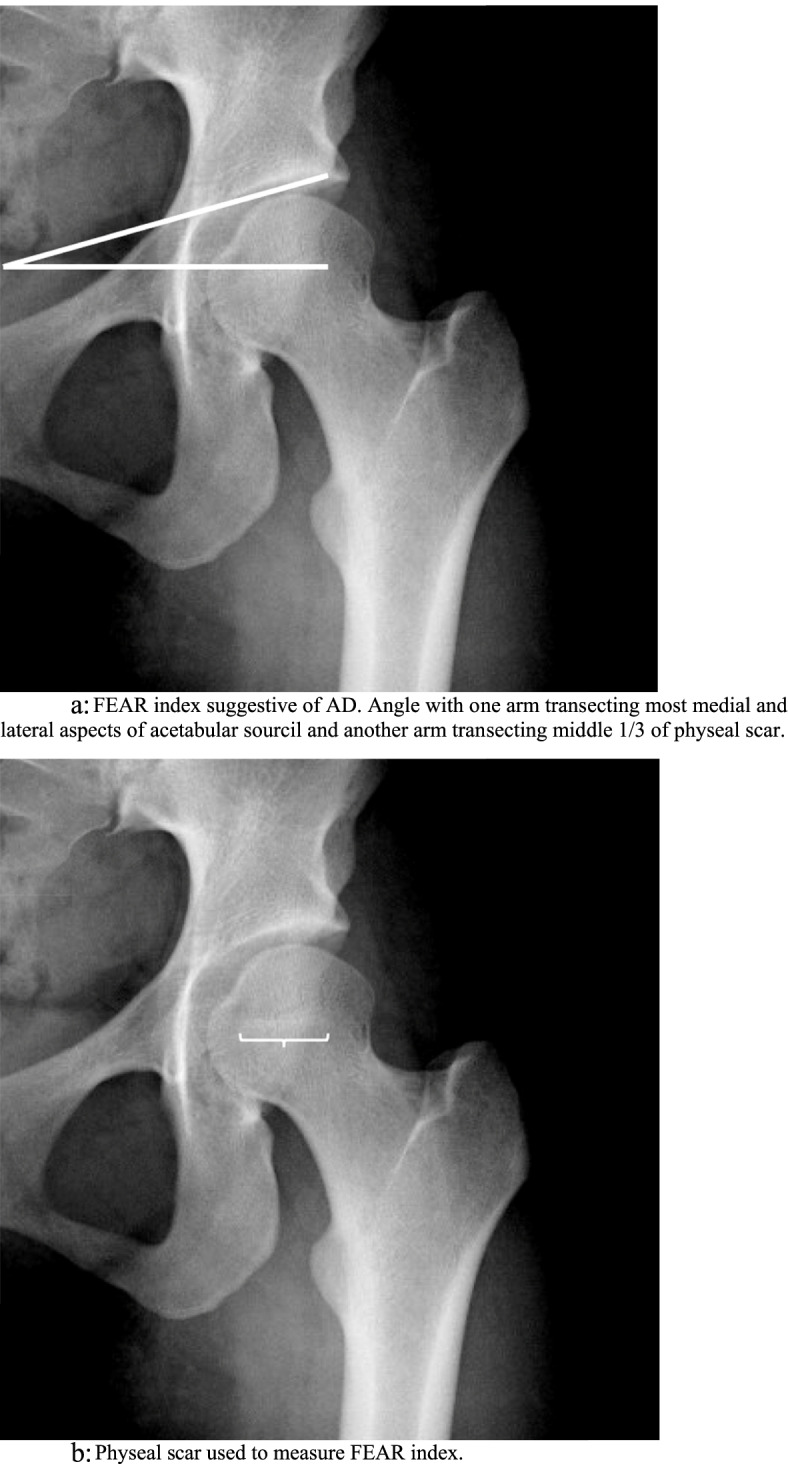
**a** FEAR index suggestive of AD. Angle with one arm transecting most medial and lateral aspects of acetabular sourcil and another arm transecting middle 1/3 of physeal scar. **b** Physeal scar used to measure FEAR index

To describe AD, the lateral center edge angle (LCEA) and anterior center edge angle (ACEA) were measured, with measures 15–25° considered as mild dysplasia, 5–15° considered as moderate dysplasia, and < 5° considered as severe dysplasia [19–21]. Figures 2 and 3 describe LCEA and ACEA measurements, respectively. The Tönnis angle was employed to characterize AD severity as well with a cutoff of 10–20° as mild dysplasia, 20–30° as moderate dysplasia, and > 30° as severe dysplasia, and Fig. 4 depicts this measurement [22]. The femoro-epiphyseal acetabular roof (FEAR) index was used to assess hip instability – defined as migration of the femoral head – and a cutoff of > 5° was determined to be likely for AD [23]. Figure 5a depicts the FEAR index, and Fig. 5b depicts the physeal scar used for measurement. These cutoffs allow for categorization of AD severity via categorical variables, permitting proper comparison and correlation analysis with categorical spinal variables. All hip measurements were assessed on the symptomatic side.

**Fig. 6:**
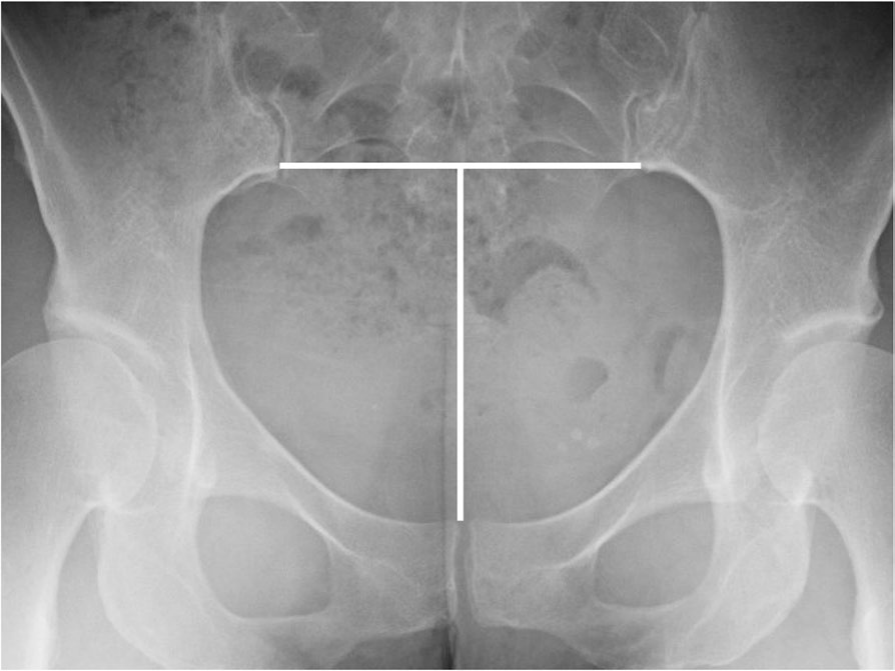
PS-SI. A line from the pubic symphysis perpendicular to a line connecting the most inferior portions of the sacroiliac joint

**Fig. 7:**
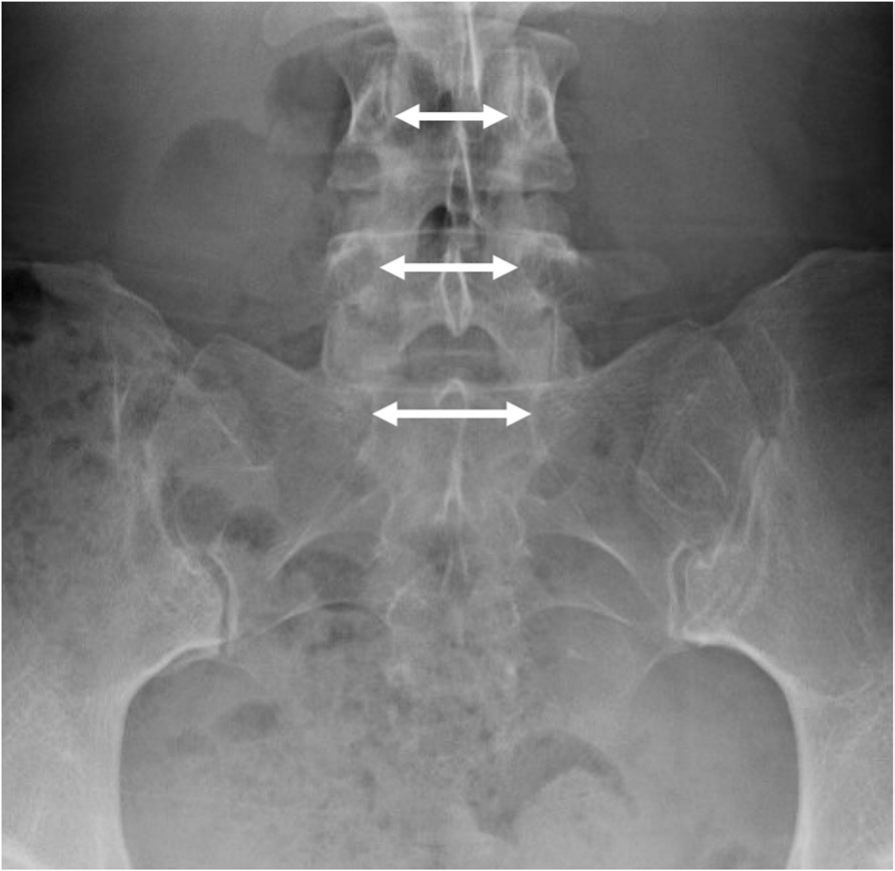
L4, L5, and L6 interpedicular distance. A line is drawn from the most medial edges of the vertebral pedicles

**Fig. 8:**
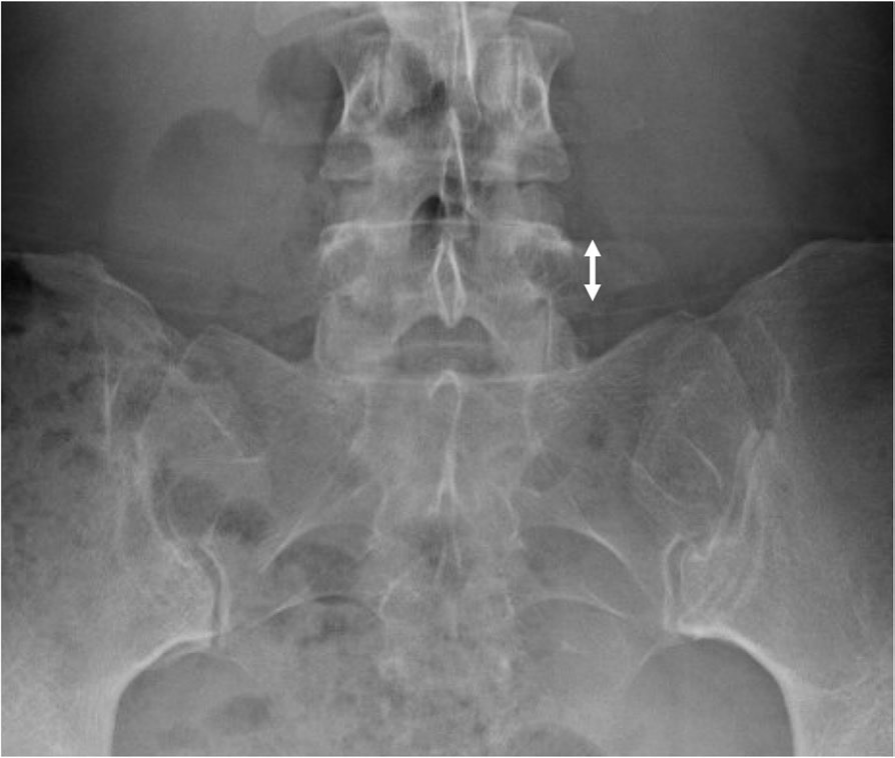
L5 transverse process height. The vertical distance between the most superior and inferior edges of L5 transverse process

**Fig. 9:**
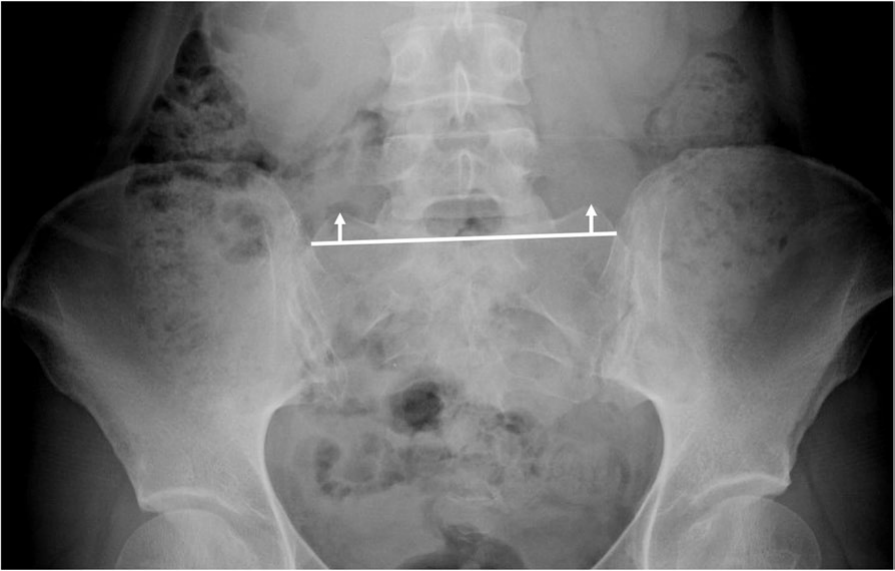
Mammillary process height. Vertical height of sacral mammillary processes perpendicular to a line connecting most superior aspects of sacroiliac joint

**Fig. 10:**
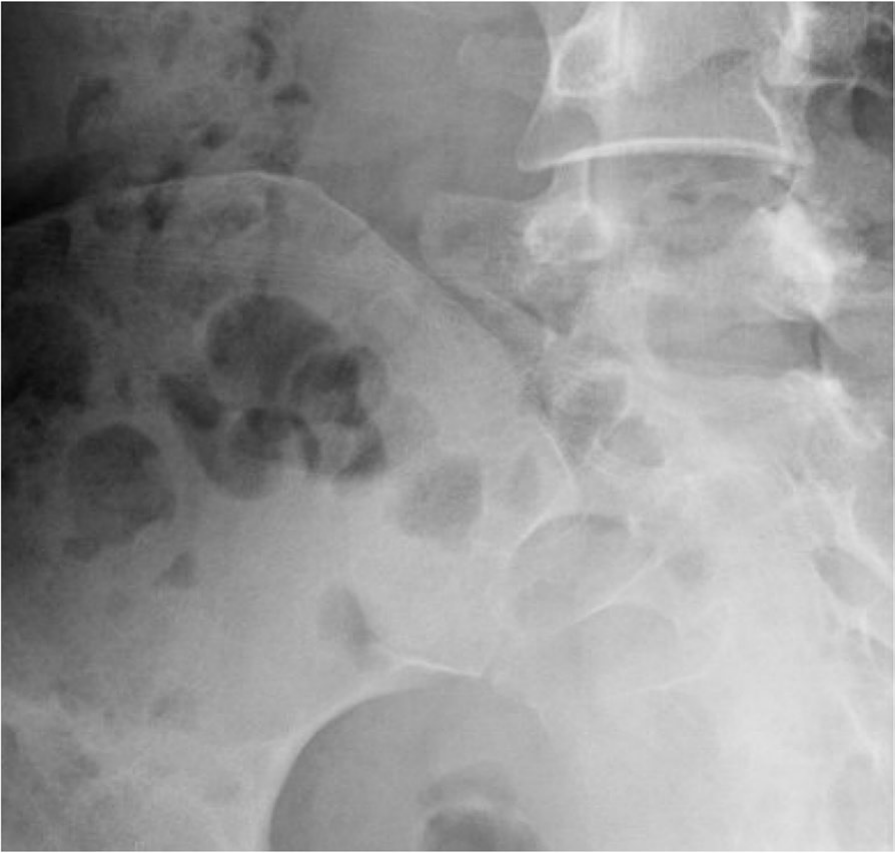
Spondylolisthesis. Anterior translocation of L5 vertebrae is appreciable on false-profile hip radiographs

**Fig. 11:**
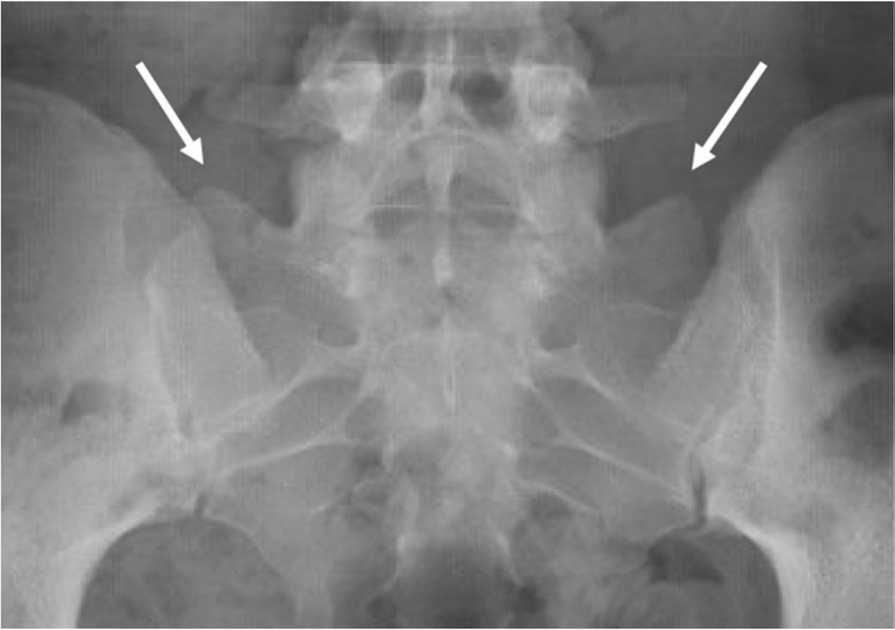
Castellvi grade 3b. Appreciable bilateral fusion of transverse processes with sacrum

To describe lumbar spine, the pubic symphysis to sacroiliac index (PS-SI) was used, an easily reproducible, validated method to characterize pelvic tilt, especially in PAO patients, which is represented by Fig. 6 [24]. The interpedicular distance at L4, L5, and S1 (Fig. 7), L5 transverse process height (Fig. 8), and mammillary process height (Fig. 9) were measured to assess for degenerative changes and fractures [25]. Spondylolisthesis with and without slippage (Fig. 10) and Castellvi classification (Fig. 11) were measured to assess for anterolisthesis and the lumbosacral transitional vertebrae and articulation [26, 27]. The false profile radiographic view used for assessment of anterior femoral head coverage also supplies an oblique view of the L5 vertebrae, permitting accurate assessment of pars interarticularis defect.

### Patient reported outcome measures

To assess patient reported outcome measures (PROMs), the Harris Hip Score (HHS) and Hip Disability and Osteoarthritis Outcome Score (HOOS) were used in the form of patient surveys to assess levels of patient-reported function and symptoms [28]. Cutoffs of HHS were determined to be < 70 as a poor result, 70–80 considered fair, 80–90 considered good, and 90–100 considered excellent for unilateral measurements [29]. Additionally, the International Hip Outcome Tool (iHOT-12) and Hip Outcome Score (HOS) were used to measure health-related quality of life [30, 31]. Pairwise correlation heat map distribution of PROMs can be seen in Fig. 1.

Electronic chart review was performed to collect basic demographic data (age, BMI, and sex). All patients received the diagnosis of AD by the senior author, a fellowship trained hip preservation orthopedic surgeon. The diagnosis was based on a combination of factors concerning lateral hip or groin pain with insidious onset, physical exam findings, and supportive radiographic evidence.

### Data analysis

Inter-rater agreement was evaluated on all hip and spine variables to ensure consistent measurements. The intraclass correlation coefficient (ICC) was used for all continuous and ordinal variables while Cohen’s Kappa was utilized for all nominal variables. The cutoffs for interpretation of agreement coefficient were defined according to standard measures with poor agreement less than 0.40, fair agreement 0.40–0.60, good agreement 0.60–0.75, and excellent agreement 0.75–1.00 [32].

To evaluate hip-spine measurements, one sample t-tests were performed to compare the mean L4, L5, and S1 interpedicular distances assessed in our patient population study to normally described distances in literature [33]. The continuous variables analyzed were the average values obtained by all independent observers. In the event of inconsistency among observers, the most senior reader’s analysis was given priority. Spearman rank correlation was utilized to determine correlation between AD severity and radiographic measurements, and all correlation coefficients were assessed against the null hypothesis of no correlation. False discovery rate (FDR) adjusted *p*-values were obtained with an adjusted *p* < 0.05 determined statistically significant. All analyses were completed in R (Vienna, Austria).

To evaluate patient reported outcome measures, regression analyses were conducted to assess correlation between radiographic evidence of lumbar pathology to functional outcomes. One sample t-tests were used to compare the means of HOOS and HHS, and Chi-square tests were utilized to compare proportional data.

## Results

### Inter-reader agreement

All variables tested showed “fair” agreement (coefficient > 0.40) at minimum with 15 of the 16 total variables demonstrating a minimum of “good” agreement (coefficient > 0.60) and 14 with a minimum of “excellent” agreement (coefficient > 0.75).

### Radiograph measurements

The two musculoskeletal radiologists identified 45 (33%) hips with lumbosacral transitional vertebrae, which were classified according to the Castellvi type classification [34]. Castellvi type 3B with bilateral fusion of the transverse process and sacrum was the most frequently identified variant determined by both readers, followed by 2A [16]. Pars interarticularis defect via identification on false profile radiograph was determined by both readers as well with the same five patients identified with pars interarticularis defects. Mean L4 interpedicular distance (IPD) was found to be 28.36 ± 3.97 mm, mean L5 IPD was 33.09 ± 3.92 mm, and mean L6 IPD was 38.93 ± 4.22 mm. This is a statistically significant increase in L4 and L5 IPD compared to the general population, consistent with our previous works [33].

These plain radiograph measurements were then compared to functional outcome measures. With respect to hip measurements, a significant correlation was observed between FEAR index and HOOS of the contralateral hip (adjusted *p* = 0.03, correlation coefficient = 0.38) and Tönnis angle of AD severity and HHS of the contralateral hip (adjusted *p* = 0.04, correlation coefficient = − 0.33). With respect to lumbosacral measurements, there was no statistically significant correlation between Castellvi grade, presence of spondylolisthesis, or L4-S1 interpedicular distance and the patient-reported outcome measures HHS or HOOS. These findings can be observed in Tables 1 and 2.

**Table 1:** Hip variables. Reported are correlation coefficient and 95% confidence interval. FEAR index and HOOS of the contralateral hip are significantly correlated (adjusted *p* = 0.03, correlation coefficient = 0.38), and Tönnis angle of AD severity and HHS of the contralateral hip are significantly correlated (adjusted *p* = 0.04, correlation coefficient = − 0.33)

Variable	HOOS	HHS (Symptomatic Hip)	HHS (Asymptomatic Hip)
**Asymptomatic hip**			
FEAR index	0.38 (0.21, 0.53)		−0.10 (− 0.28, 0.09)
L5 Transverse Process Height	0.31 (0.13, 0.47)		0.24 (0.05, 0.40)
LCEA	−0.02 (− 0.21, 0.16)		0.25 (0.07, 0.42)
Mammillary Process Height	−0.10 (− 0.28, 0.09)		0.06 (− 0.13, 0.24)
Tönnis angle	0.03 (−0.15, 0.22)		−0.33 (− 0.49,-0.16)
ACEA	− 0.09 (− 0.27, 0.10)		0.27 (0.08, 0.43)
**Symptomatic hip**			
FEAR index	0.31 (0.14, 0.47)	0.13 (−0.05, 0.31)	
L5 Transverse Process Height	0.14 (−0.04, 0.32)	0.11 (−0.08, 0.29)	
LCEA	−0.16 (− 0.33, 0.03)	−0.03 (− 0.21, 0.16)	
Mammillary Process Height	−0.18 (− 0.36, 0.00)	−0.11 (− 0.29, 0.08)	
Tönnis angle	0.13 (−0.05, 0.31)	− 0.03 (− 0.22, 0.15)	
ACEA	− 0.11 (− 0.29, 0.08)	−0.04 (− 0.22, 0.15)

**Table 2:** Spine variables. Reported are correlation coefficients and 95% confidence intervals. No spine variables studied are significantly correlated with outcome scores

Variable	HOOS	HHS (symptomatic hip)	HHS (asymptomatic hip)
**Castellvi**	0.07 (− 0.11, 0.26)	0.00 (− 0.19, 0.18)	0.04 (− 0.15, 0.22)
**Spondylolisthothesis**	−0.12 (− 0.30, 0.07)	−0.17 (− 0.34, 0.02)	−0.26 (− 0.42,-0.08)
**PS-SI**	− 0.19 (− 0.37,-0.01)	−0.02 (− 0.20, 0.17)	−0.03 (− 0.22, 0.15)
**L4 interpedicular distance**	0.31 (0.13, 0.47)	0.20 (0.01, 0.37)	0.13 (− 0.05, 0.31)
**L5 interpedicular distance**	0.11 (−0.08, 0.29)	0.10 (− 0.09, 0.28)	0.23 (0.05, 0.40)
**S1 interpedicular distance**	−0.08 (− 0.26, 0.11)	0.04 (− 0.15, 0.22)	0.19 (0.00, 0.36)

## Discussion

This is the first study to report the association between lumbosacral anomalies and patient-reported outcome measures. Although we hypothesized that AD patients with radiographic evidence of lumbar spine anomalies are associated with decreased function in comparison to AD patients without such radiographic findings, our results demonstrate no significant correlation for Castellvi grade, presence of spondylolisthesis, or IPD. We have previously found that AD patients are associated with increased IPD [16]. This can be explained from the increased mechanical stress in the lumbosacral spine from dysplastic transverse processes with extra-foraminal stenosis, resulting in widened lumbosacral pedicles [35]. However, it appears this increased IPD is not associated with decreased patient function. This is an intriguing result which points toward the nature of these spinal anomalies and helps guide treatment: presence and severity of lumbosacral pathology does not necessitate surgical treatment for AD, as patient function is preserved. A possible explanation includes the compensation by adjacent musculoskeletal structures.

Furthermore, this study reveals the unreliability of utilizing hip radiographs to determine function. This is the first study to report the association between radiographic measures of AD and the outcome measures HHS and HOOS. Although we hypothesized that those with decreased LCEA, ACEA, and FEAR index or increased Tönnis angle of AD severity would be correlated with decreased HHS or HOOS, we found no statistically significant correlation for the diseased ipsilateral hip. This finding reflects the importance of compensatory mechanisms in patients with AD: it is well documented that anterior pelvic tilt is increased in similar conditions like FAI, and this anterior pelvic tilt may allow for improved patient mobility and function increased axial load on the lumbar spine [8]. Additionally, interreader agreement evaluation of plain radiographs is strong in our study, as all 16 variables studied showed at least “fair” agreement (coefficient > 0.4) with 14 demonstrating “excellent” agreement (coefficient > 0.75). Therefore, the severity of AD should not be determined solely from plain radiographs but in combination with clinical findings.

There are several limitations of this study. Firstly, not all patients with AD could be included in the study due to missing full hip x-ray series. Additionally, the results of our study are limited by the retrospective nature of data review. All patients were gathered from a single academic institution, which may not represent the general population. Another possible limitation of this study is a lack of measurements of the sacroiliac joint, which may factor into the complexity of the hip-spine relationship. In the future, studies assessing outcomes after surgical treatment of AD can help characterize the effect of surgery on patient function. Such operations to evaluate include PAO, THA, or spine surgery. Another study would be to assess the correlation between these radiographic anomalies and patient-reported pain location, as it is well documented that patients with AD experience pain in the lateral hip, groin, lower back, thigh, knee, and buttocks [36]. Another possible extension of this study would be to analyze advanced imaging such as lumbar spine MRI and CT instead of radiographs for greater sensitivity of measurements. These studies may provide further insight into the utility of PROMs for measuring functional status and pain levels in AD patients both preoperatively and postoperatively.

## Data Availability

The datasets used and/or analyzed during the current study are not publicly available due to individual privacy but are available from the corresponding author on reasonable request.
